# Trajectory-based profiling of 52-week secukinumab response reveals baseline clinical and transcriptomic differences in plaque psoriasis

**DOI:** 10.3389/fimmu.2026.1902716

**Published:** 2026-07-31

**Authors:** Xiuxiu Wang, Ling Han, Jing Li, Yuliang Rao, Shujie Zhang, Ning Yu

**Affiliations:** 1 Department of Dermatology, Huashan Hospital, Fudan University, Shanghai, China; 2 Eye Institute and Department of Ophthalmology, the Eye & ENT Hospital, Fudan University, Shanghai, China; 3 National Experimental Teaching Demonstration Center of Basic Medicine, Fudan University, Shanghai, China

**Keywords:** latent class mixed model, psoriasis, secukinumab, transcriptomics, treatment trajectory

## Abstract

**Background:**

Response to secukinumab in psoriasis varies over time and is not fully captured by single timepoint endpoints. Baseline features associated with long-term response trajectories remain unclear.

**Objective:**

To identify 52-week secukinumab response trajectories and examine baseline clinical and peripheral blood mononuclear cell (PBMC) transcriptomic features associated with divergent trajectories.

**Methods:**

We analyzed 485 secukinumab-treated patients with plaque psoriasis from the Shanghai Psoriasis Effectiveness Evaluation CoHort (SPEECH). Latent class mixed models were used to derive 52-week Psoriasis Area and Severity Index (PASI) trajectories. Baseline factors associated with the suboptimal trajectory were assessed by logistic regression. In an exploratory subset of 27 patients, paired PBMC samples collected at baseline and week 4 underwent bulk RNA sequencing.

**Results:**

Two PASI trajectories were identified: an optimal trajectory (432/485, 89.1%) with rapid, sustained improvement and a suboptimal trajectory (53/485, 10.9%) with higher disease activity. Higher baseline PASI, body mass index (BMI), prior biologic exposure, multi-seasonal flare pattern, and infection-triggered flare history were independently associated with the suboptimal trajectory. Week 4 PASI improvement showed moderate discrimination between the two groups. Baseline PBMC transcriptomes also differed, with the optimal trajectory showing a more prominent mature low-density neutrophil signature and a greater reduction in that signature from baseline to week 4.

**Conclusion:**

Long-term response to secukinumab followed two distinct trajectories. Baseline PASI, BMI, prior biologic exposure, patient-reported flare history, and the mature low-density neutrophil signature may help identify patients with different long-term response patterns.

## Introduction

Psoriasis is a chronic, immune-mediated inflammatory skin disease with substantial heterogeneity in severity, clinical course, environmental triggers, and treatment response (1–3). Biologics targeting the IL-23/IL-17 axis have markedly improved outcomes for patients with moderate-to-severe plaque psoriasis (4). Secukinumab, a fully human monoclonal antibody against IL-17A, has shown high efficacy in both randomized trials and real-world settings (5, 6). Nevertheless, response during long-term treatment remains variable. Some patients achieve rapid and sustained skin clearance, while others experience delayed improvement, incomplete response, or loss of effectiveness over time.

Treatment response in psoriasis is commonly assessed using cross-sectional endpoints, such as Psoriasis Area and Severity Index (PASI) 90, PASI 100, or Physician Global Assessment (PGA) 0/1 at week 12 or week 52. Although clinically useful, these fixed timepoint measures do not fully describe the longitudinal pattern of response (7). Trajectory-based approaches provide an alternative framework by identifying latent subgroups of patients who follow distinct response patterns over time (8–11). This may be particularly useful in real-world settings, where treatment courses are heterogeneous and some patients remain at risk of persistently higher disease activity despite biologic therapy.

Identifying baseline factors associated with favorable or unfavorable long-term response patterns may help guide treatment decisions. Previous studies have suggested that obesity, prior biologic exposure, sex, and disease duration are associated with biologic treatment outcomes at specific timepoints (12–15). However, less is known about whether these or other baseline features are related to trajectory-based long-term treatment outcomes.

The biological basis of divergent treatment trajectories also remains unclear. Peripheral blood mononuclear cell (PBMC) transcriptomic profiling offers a way to examine whether patients who subsequently follow different clinical courses already differ in their systemic inflammatory state before treatment (16). Paired early samples may further reveal molecular changes that accompany the initial response to IL-17A blockade (17). Such analyses are relevant because psoriasis is increasingly recognized as a systemic inflammatory disease rather than a condition limited to the skin.

In this study, we used latent class mixed models (LCMMs) to identify 52-week PASI response trajectories among 485 patients with plaque psoriasis treated with secukinumab in the Shanghai Psoriasis Effectiveness Evaluation CoHort (SPEECH). We then examined baseline clinical factors associated with the suboptimal trajectory. In an exploratory subset with paired PBMC samples collected at baseline and week 4, we performed bulk RNA sequencing to investigate baseline transcriptomic differences and early treatment-induced molecular changes associated with subsequent clinical trajectory.

## Materials and methods

### Data source and study population

Data were derived from the SPEECH registry, an ongoing multicenter observational cohort conducted at seven dermatology departments in Shanghai, China (18, 19). The registry received institutional review board approval from all participating sites in 2021, covering the present analysis, and all participants provided written informed consent. The study was conducted and reported in accordance with the Strengthening the Reporting of Observational Studies in Epidemiology (STROBE) guidelines. Between March 2021 and June 2023, patients with psoriasis initiating biologic therapy were enrolled in the SPEECH registry. For the present analysis, we included 485 adult patients with plaque psoriasis who initiated secukinumab treatment and had complete baseline data together with at least one post-baseline PASI assessment. Eligible patients were aged ≥ 18 years, had a baseline PASI score ≥ 5, and received secukinumab as monotherapy during follow-up, without concomitant systemic or topical psoriasis treatment. Patients were excluded if they had received oral systemic therapy or phototherapy within 4 weeks before enrollment, or biologic therapy within 12 weeks before enrollment.

### Latent class mixed models

Latent class mixed models (LCMMs) were fitted to identify distinct longitudinal PASI response patterns during 52 weeks of secukinumab treatment (20). PASI was specified as the outcome variable and time since treatment initiation as the longitudinal predictor. To reduce skewness and improve model convergence, PASI values were transformed as log(PASI + 1). A random intercept was included to account for within-patient correlation across repeated measurements, and class-specific fixed effects of time were specified to allow different trajectory shapes across latent classes. Models with one to five classes were evaluated. The optimal number of classes was selected according to the Bayesian Information Criterion (BIC), clinical interpretability, and a minimum class size of 5% of the study population. Posterior probabilities were calculated for each model, and patients were assigned to the class with the highest posterior probability. Trajectory modeling was performed using longitudinal PASI measurements from baseline to week 52. LCMMs were fitted using the *lcmm* package in R.

### Multiple imputation

Missing longitudinal PASI data were addressed using multiple imputation by chained equations under the missing-at-random assumption. The imputation model included baseline demographic and clinical characteristics together with all available PASI measurements across follow-up visits. A total of 100 imputed datasets were generated. Subsequent trajectory analyses were performed separately in each imputed dataset. For each patient, posterior probabilities of class membership were obtained from the fitted latent class mixed models and averaged across imputations to derive pooled posterior probabilities. Patients were then assigned to the trajectory class with the higher pooled posterior probability. MI was performed in R using the *mice* package.

### Baseline characteristics

Baseline characteristics were collected at treatment initiation, including demographics, disease history, prior treatment exposure, comorbidities, joint symptoms, patient-reported flare seasonality, trigger history, and disease severity. Disease severity was assessed using PASI and Dermatology Life Quality Index (DLQI). Comorbidity burden was defined as the number of the following conditions: cardiovascular disease, diabetes, hypertension, non-alcoholic fatty liver disease (NAFLD), hyperlipidemia, and hyperuricemia. Flare seasonality was classified as aseasonal, multi-seasonal, winter flare, or summer flare. Trigger history was based on patient report. Infection-triggered flare history was defined as psoriasis worsening within 1 month after infection, including upper respiratory infection or periodontitis; stress-triggered flare history was recorded separately. Baseline characteristics were summarized by PASI trajectory group. Continuous variables are presented as median with interquartile range, and categorical variables are presented as number and percentage.

### Identification of clinical predictors

Univariable and multivariable logistic regression analyses were performed to identify baseline factors associated with the suboptimal PASI response trajectory. Odds ratios (ORs) and 95% confidence intervals (CIs) were estimated for each variable. Candidate predictors included demographic characteristics, baseline disease severity, treatment history, comorbidity-related variables, and patient-reported flare features, including flare seasonality pattern and trigger history. Because the number of patients in the suboptimal trajectory group was limited, variable selection for the multivariable model was restricted to clinically relevant factors selected *a priori* and variables associated with the outcome at *P* < 0.05 in univariable analysis. Flare seasonality pattern and trigger history were evaluated as baseline predictors in the multivariable model. Results of the final model are presented as adjusted ORs (aORs) with 95% CIs. Model fit was assessed using the Hosmer–Lemeshow goodness-of-fit test.

### Early week 4 clinical response analysis

Week 4 was selected as an early exploratory assessment point because it represented the earliest post-baseline visit with paired clinical and transcriptomic data, allowing us to examine whether divergence between long-term trajectories was already detectable within the first month of treatment. This analysis was intended to complement, rather than replace, conventional later clinical benchmarks. Percentage PASI improvement at week 4 was calculated for each patient as follows: PASI improvement at week 4 = (baseline PASI – week 4 PASI)/baseline PASI. Percentage PASI improvement at week 4 was compared between the optimal and suboptimal PASI trajectory groups. Receiver operating characteristic (ROC) analysis was then performed to assess the ability of week 4 PASI improvement to distinguish between the two trajectory classes. The area under the curve (AUC) and its 95% CI were calculated. The optimal cut-off value was determined using the Youden index.

### PBMC isolation

Peripheral blood was collected into heparinized tubes. PBMCs were isolated by Ficoll/Hypaque density-gradient centrifugation according to standard procedures. After isolation, the PBMC fraction was washed and resuspended in EasySep buffer (Stemcell Technologies, Vancouver, Canada). Cells were immediately lysed in TRIzol reagent (Invitrogen, Shanghai, China) and stored at −80 °C until RNA extraction for bulk RNA sequencing.

### RNA sequencing

As previously reported (21), total RNA was extracted from PBMC samples using TRIzol reagent according to the manufacturer’s instructions. RNA concentration and purity were assessed using a NanoDrop 2000 spectrophotometer (Thermo Fisher Scientific, Shanghai, China), and RNA integrity was evaluated using an Agilent 2100 Bioanalyzer (Agilent Technologies, Santa Clara, CA, USA). Sequencing libraries were prepared using the TruSeq Stranded mRNA LT Sample Prep Kit (Illumina, San Diego, CA, USA) and sequenced on the Illumina HiSeq X Ten platform to generate 150-bp paired-end reads.

Raw FASTQ reads were processed using Trimmomatic for adaptor trimming and quality filtering. Clean reads were aligned to the human reference genome (GRCh38) using HISAT2. Gene expression abundance was quantified as fragments per kilobase of transcript per million mapped reads using Cufflinks, and gene-level read counts were generated using HTSeq-count for downstream differential expression analysis.

### Bioinformatic analysis

Bioinformatic analyses were performed in R using gene-level count matrices derived from bulk PBMC RNA sequencing. For baseline analyses, pretreatment transcriptomic profiles were compared between patients who were subsequently classified into the optimal (n = 16) and suboptimal (n = 11) PASI response trajectory groups.

Principal component analysis (PCA) was performed using expressed genes after normalization. Hierarchical clustering was performed using the 1000 genes with the highest variance across samples. Differential expression analysis was performed using DESeq2. For baseline comparisons, gene expression was compared between the optimal and suboptimal trajectory groups. For early treatment-response analyses, paired week 4 and baseline samples were compared within each trajectory group using a paired design with patient identifier included in the model.

*P* values were adjusted for multiple testing using the Benjamini–Hochberg method. Differentially expressed genes (DEGs) were defined as those with an adjusted *P* value < 0.05 and |log_2_ fold change| ≥ 1 for baseline comparisons, or |log_2_ fold change| ≥ 0.58 for week 4 versus baseline comparisons. Gene Ontology analysis was performed using the Gene Ontology immune system process ontology set in ClueGO, version 2.5.10, within Cytoscape, version 3.10.4.

Pathway activity was inferred using PROGENy based on downstream target gene expression patterns. Pathway scores were compared between trajectory groups at baseline. Sample-wise enrichment scores for Hallmark pathways and immune cell signatures were calculated using gene set variation analysis (GSVA) (21, 22). All immune cell gene sets used for scoring are provided in **Supplementary Dataset 1**. To assess early changes in low-density neutrophil transcriptional activity, enrichment scores for the low-density neutrophil signature were calculated in paired baseline and week 4 PBMC samples. For each patient, the change in enrichment score was defined as the week 4 score minus the baseline score. Changes in enrichment score were compared between the optimal and suboptimal trajectory groups. ROC analysis was performed to evaluate the ability of this change to distinguish between the two trajectory classes.

## Results

### Secukinumab-treated patients followed two distinct 52-week PASI response trajectories

**Figure 1:**
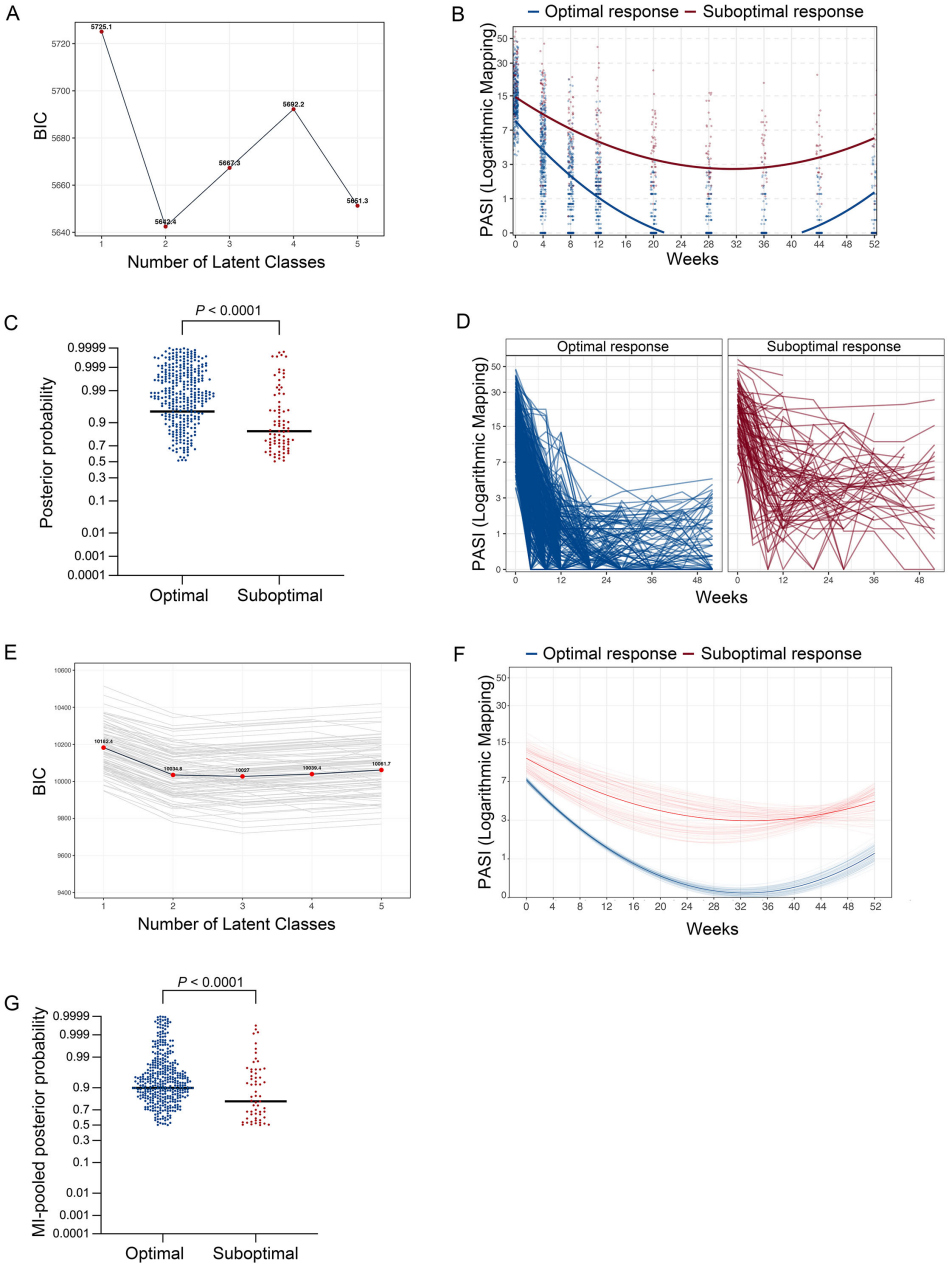
PASI response trajectories identified by latent class mixed modeling before and after multiple imputation. **(A)** Bayesian information criterion (BIC) values for latent class mixed models with one to five classes. **(B)** Estimated 52-week PASI trajectories for the optimal and suboptimal response groups. **(C)** Posterior probability distributions for class assignment in the two trajectory groups. Horizontal bars indicate group medians. **(D)** Individual longitudinal PASI profiles in the two trajectory groups. **(E)** BIC values for models with one to five latent classes across 100 multiply imputed datasets. Gray lines represent individual imputed datasets, and red points indicate pooled consensus BIC values. **(F)** MI-pooled 52-week PASI trajectories in the two trajectory groups. Thin lines represent trajectories from individual imputed datasets, and bold lines represent pooled trajectories. **(G)** MI-pooled posterior probability distributions for class assignment in the two trajectory groups. Horizontal bars indicate group medians.

Latent class mixed modeling was performed using the observed longitudinal PASI data from 485 eligible patients to identify 52-week response patterns to secukinumab. Model selection based on the BIC supported a two-class solution (**Figure 1A**). The estimated trajectories showed an optimal response group with rapid and sustained improvement to near-complete skin clearance, as well as a suboptimal response group with persistently higher PASI during follow-up (**Figure 1B**). Posterior probability distributions indicated good separation between the two classes. Posterior probabilities were generally higher in the optimal trajectory group than in the suboptimal trajectory group, suggesting more stable class assignment in the optimal group and greater heterogeneity within the suboptimal trajectory (**Figure 1C**). Individual longitudinal plots further showed relatively homogeneous improvement in the optimal trajectory, whereas greater inter-individual variability was observed in the suboptimal trajectory (**Figure 1D**).

To account for missing PASI data during follow-up, the analysis was repeated across 100 MI datasets. The BIC again supported a two-class solution, with both the individual imputed BIC curves and the pooled consensus BIC showing an elbow at two classes (**Figure 1E**). The MI-pooled trajectories were consistent with the non-imputed analysis, showing optimal and suboptimal response groups (**Figure 1F**). MI-pooled posterior probability distributions also indicated good class separation, with median probabilities above 0.8 in both trajectory groups (**Figure 1G**), supporting the stability of the two-class solution.

Among the 485 patients, 432 (89.1%) were assigned to the optimal response trajectory and 53 (10.9%) to the suboptimal response trajectory. Baseline characteristics stratified by PASI response trajectory, based on the multiple imputation-based consensus classification, are presented in **Table 1**.

**Table 1:** Baseline characteristics of patients stratified by PASI response trajectories defined using the multiple imputation-based consensus classification.

Characteristics	Entire cohort	Optimal response trajectory	Suboptimal response trajectory
Patients, n (%)	485	432 (89.1)	53 (10.9)
Age, years	44 (35–58)	44 (34–59)	43 (36–56)
Disease duration, years	13 (8–20)	13 (8–20)	13 (8–22)
Male sex, n (%)	367 (75.7)	320 (74.1)	47 (88.7)
College education, n (%)	314 (64.7)	282 (65.3)	32 (60.4)
BMI (kg/m^2^)	24.8 (22.6–27.3)	24.6 (22.5–26.8)	26.2 (23.9–30.9)
Ever smoked, n (%)	196 (40.4)	169 (39.1)	27 (50.9)
Family history of psoriasis, n (%)	116 (23.9)	108 (25.0)	8 (15.1)
Prior biologic exposure, n (%)	67 (13.8)	52 (12.0)	15 (28.3)
Prior conventional systemic therapy, n (%)	204 (42.1)	175 (40.5)	29 (54.7)
Prior phototherapy, n (%)	231 (47.6)	201 (46.5)	30 (56.6)
Psoriatic arthritis, n (%)	45 (9.3)	40 (9.3)	5 (9.4)
Number of joint symptoms^a^, n (%)			
0	404 (83.3)	364 (84.3)	40 (75.5)
1	65 (13.4)	53 (12.3)	12 (22.6)
≥ 2	16 (3.3)	15 (3.5)	1 (1.9)
Number of comorbidities^b^, n (%)			
0	305 (62.9)	274 (63.4)	31 (58.5)
1	101 (20.8)	90 (20.8)	11 (20.8)
≥ 2	79 (16.3)	68 (15.7)	11 (20.8)
Flare seasonality pattern^c^, n (%)			
Aseasonal	120 (24.7)	109 (25.2)	11 (20.8)
Multi-seasonal flare	91 (18.8)	75 (17.4)	16 (30.2)
Winter flare	241 (49.7)	219 (50.7)	22 (41.5)
Summer flare	33 (6.8)	29 (6.7)	4 (7.5)
Trigger history^d^, n (%).			
Infection-triggered flare history^e^	101 (20.8)	82 (19.0)	19 (35.8)
Stress-triggered flare history	50 (10.3)	48 (11.1)	2 (3.8)
Baseline PASI	14.2 (10.4–20.6)	13.6 (10.2–19.6)	20.5 (13.6–28.1)
Baseline DLQI	10 (6–17)	10 (6–16)	13 (8–18)

PASI, Psoriasis Area and Severity Index; BMI, body mass index; DLQI, Dermatology Life Quality Index.

Continuous variables are presented as median (interquartile range) and categorical variables are presented as number (percentage).

^a^ Joint symptoms included joint pain, digit pain, heel pain, back pain and stiffness.

^b^ Comorbidities included cardiovascular disease, diabetes, hypertension, non-alcoholic fatty liver disease (NAFLD), hyperlipidemia, and hyperuricemia.

^c^ Mutually exclusive.

^d^ Multiple responses allowed.

^e^ Infection-triggered flares referred to psoriasis exacerbation within 1 month after infection; the main specified infection-related triggers were upper respiratory tract infection and periodontitis.

### Higher baseline PASI, BMI, prior biologic exposure, and flare features were associated with the suboptimal trajectory

Univariable and multivariable logistic regression analyses were then performed to identify baseline factors associated with the suboptimal PASI response trajectory (**Table 2**). In univariable analysis, male sex, higher body mass index (BMI), prior biologic exposure, prior conventional systemic therapy, multi-seasonal flare pattern, infection-triggered flare history, and higher baseline PASI were associated with greater odds of the suboptimal trajectory. In the multivariable model, higher baseline PASI, higher BMI, prior biologic exposure, multi-seasonal flare pattern, and infection-triggered flare history remained associated with the suboptimal trajectory.

**Table 2:** Logistic regression analysis of factors associated with suboptimal PASI response trajectories.

Variables	Univariable analysis		Multivariable analysis	
	OR (95% CI)	*P* value	OR (95% CI)	*P* value
Age, per 1 year increase	1.00 (0.98–1.02)	0.977		
Disease duration, per 1 year increase	1.01 (0.98–1.04)	0.448	1.01 (0.98–1.04)	0.530
Male sex	2.69 (1.19–6.07)	0.018	2.06 (0.83–5.13)	0.121
College education	0.66 (0.38–1.13)	0.132		
BMI, per 1 kg/m^2^ increase	1.15 (1.07–1.22)	<0.001	1.12 (1.04–1.20)	0.002
Ever smoked	1.59 (0.93–2.73)	0.093		
Family history of psoriasis	0.67 (0.34–1.34)	0.257		
Prior biologic exposure	2.91 (1.55–5.47)	0.001	3.09 (1.51–6.35)	0.002
Prior conventional systemic therapy	1.86 (1.08–3.20)	0.026		
Prior phototherapy	1.53 (0.89–2.64)	0.124		
Psoriatic arthritis	1.09 (0.44–2.69)	0.852		
Number of joint symptoms^a^, per 1 increase	1.41 (0.86–2.32)	0.177		
Number of comorbidities^b^, per 1 increase	1.05 (0.79–1.39)	0.729		
Flare seasonality pattern, ref: aseasonal				
Multi-seasonal flare	2.30 (1.05–5.05)	0.038	2.42 (1.02–5.75)	0.045
Winter flare	1.12 (0.54–2.30)	0.763	1.13 (0.51–2.47)	0.769
Summer flare	1.28 (0.38–4.25)	0.689	1.36 (0.37–4.96)	0.646
Trigger history				
Infection-triggered flare history^c^	2.40 (1.35–4.27)	0.003	2.24 (1.16–4.31)	0.016
Stress-triggered flare history	0.28 (0.07–1.20)	0.087	0.25 (0.06–1.11)	0.068
Baseline PASI	1.07 (1.04–1.10)	<0.001	1.07 (1.04–1.10)	<0.001
Baseline DLQI	1.02 (0.98–1.06)	0.255		

PASI, Psoriasis Area and Severity Index; OR, odds ratio; CI, confidence interval; BMI, body mass index.

^a^ Joint symptoms included joint pain, digit pain, heel pain, back pain and stiffness.

^b^ Comorbidities included cardiovascular disease, diabetes, hypertension, non-alcoholic fatty liver disease (NAFLD), hyperlipidemia, and hyperuricemia.

^c^ Infection-triggered flares referred to psoriasis exacerbation within 1 month after infection; the main specified infection-related triggers were upper respiratory tract infection and periodontitis.

### Week 4 PASI improvement was already aligned with subsequent long-term response trajectories

**Figure 2:**
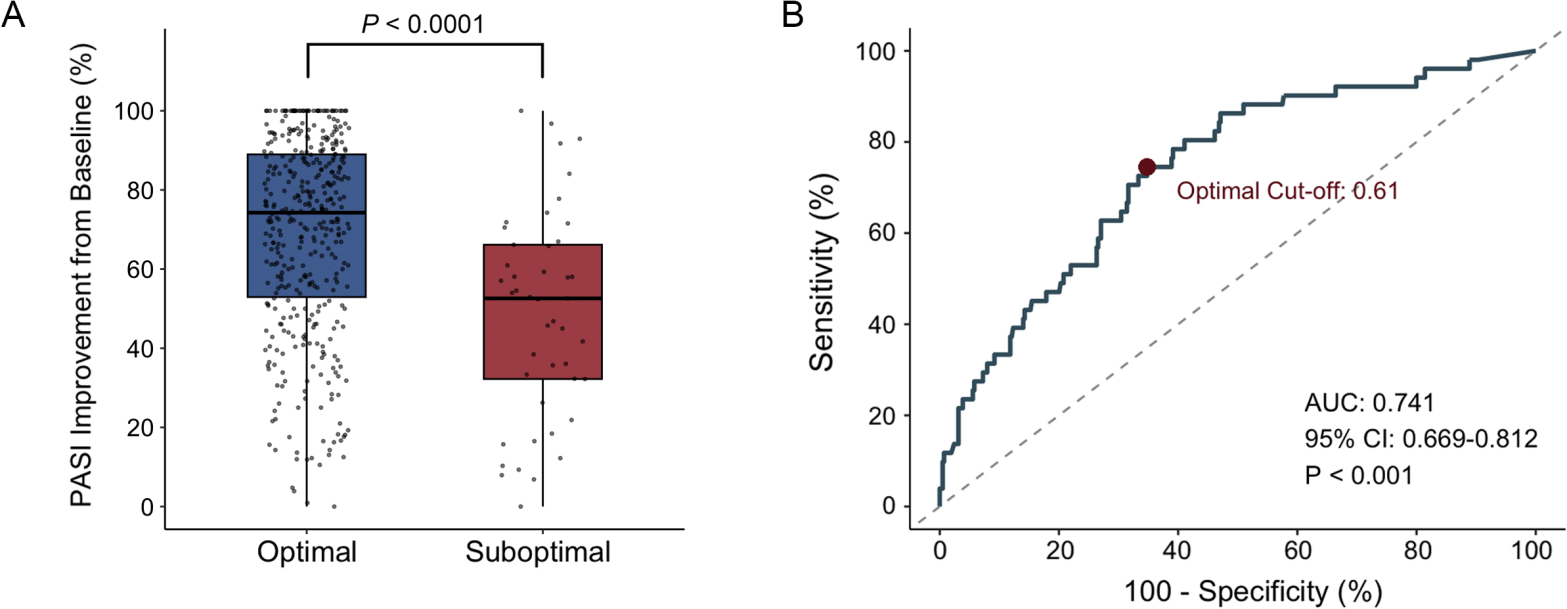
Early week 4 clinical response according to PASI trajectory classification. **(A)** Percentage PASI improvement from baseline to week 4 in the optimal and suboptimal trajectory groups. Boxes indicate the interquartile range, center lines indicate medians, and whiskers indicate the full range. Individual patients are shown as dots. *P* values were calculated using the Wilcoxon rank-sum test. **(B)** Receiver operating characteristic curve of week 4 PASI improvement for distinguishing the two trajectory groups. The area under the curve (AUC), 95% confidence interval, and optimal cut-off value are shown.

To determine whether long-term trajectory divergence could already be detected early after treatment initiation, we compared clinical response at week 4 between the two trajectory groups. Week 4 PASI improvement was greater in the optimal trajectory group than in the suboptimal trajectory group (**Figure 2A**). ROC analysis showed that early clinical response at week 4 discriminated between the two groups with moderate accuracy (AUC 0.741, 95% CI 0.669–0.812; *P* < 0.001) (**Figure 2B**). The optimal cut-off was 61%, indicating that patients with greater early improvement were more likely to follow the optimal 52-week trajectory.

### Baseline PBMC transcriptomes differed between patients with optimal and suboptimal trajectories

**Figure 3:**
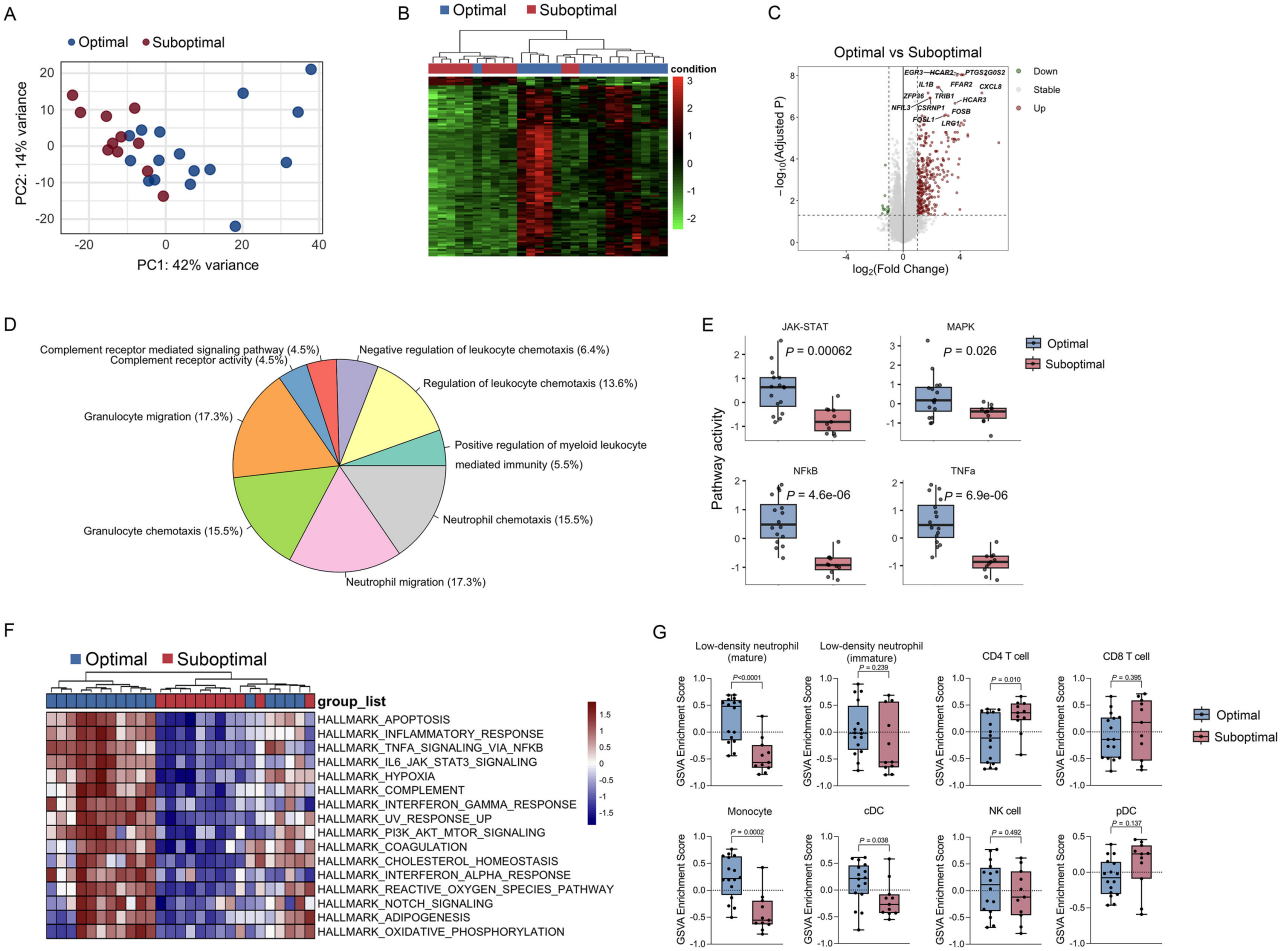
Baseline PBMC transcriptomic differences according to subsequent PASI trajectory. **(A)** PCA of the transcriptome data in PBMCs from patients in optimal (n = 16) and suboptimal (n = 11) trajectory groups. **(B)** Hierarchical clustering of the 1000 genes with the highest variance within the dataset, shown as an unsupervised visualization of transcriptomic structure. **(C)** Volcano plot showing DEGs between the optimal and suboptimal trajectory groups at baseline. Genes with an |log_2_ fold change| ≥ 1 and an adjusted *P* < 0.05 were considered significant. **(D)** GO analysis of optimal-up DEGs using the GO immune system process category. Categories shown are those reaching statistical significance using a two-sided hypergeometric test. **(E)** PROGENy pathway activity scores. **(F)** GSVA enrichment heatmap shown with unsupervised clustering for visualization. **(G)** GSVA enrichment scores for immune cell signatures in the two trajectory groups.

To investigate baseline systemic inflammatory features associated with subsequent long-term clinical trajectories, bulk RNA sequencing was performed on pretreatment PBMC samples from 27 patients in the cohort. These patients were subsequently classified into the optimal (n = 16) or suboptimal (n = 11) trajectory groups, and their baseline characteristics are shown in **Supplementary Table 1**. PCA and hierarchical clustering showed separation between the two trajectory groups at baseline (**Figures 3A, B**). Notably, the heatmap panels were intended as unsupervised visual summaries, while all differential expression analyses were performed according to the clinically defined trajectory groups.

Baseline PBMC gene expression was then compared between the optimal and suboptimal trajectory groups. This identified 346 DEGs, including 333 genes expressed at higher levels and 13 genes expressed at lower levels in the optimal trajectory group compared with the suboptimal trajectory group (|log_2_ fold change| ≥ 1, adjusted *P* < 0.05; **Supplementary Dataset 2**). Among the genes expressed at higher levels in the optimal trajectory group were several genes related to innate myeloid inflammation and early activation responses (**Figure 3C**).

Gene Ontology analysis of genes expressed at higher levels in the optimal trajectory group identified nine immune system process terms, mainly related to neutrophil-mediated immunity (**Figure 3D**). PROGENy analysis indicated higher baseline activity of several inflammatory and growth-related pathways in the optimal trajectory group, including TNF-α, NF-κB, JAK-STAT, and MAPK signaling (**Figure 3E**). Hallmark pathway analysis using GSVA further showed higher enrichment of inflammatory programs, including TNF-α signaling via NF-κB, inflammatory response, and IL-6/JAK-STAT3 signaling, in the optimal trajectory group (**Figure 3F**). GSVA based on immune cell signatures showed higher enrichment of mature low-density neutrophils, monocytes, and conventional dendritic cells in the optimal trajectory group (**Figure 3G**).

### Early transcriptomic changes after secukinumab treatment were more pronounced in the optimal trajectory group

To examine early molecular changes associated with subsequent 52-week clinical trajectories, paired PBMC transcriptomes at baseline and week 4 were compared within each trajectory group. The DEGs from the week 4 versus baseline comparisons in the optimal and suboptimal trajectory groups are listed in **Supplementary Datasets 3** and **Supplementary Dataset 4**, respectively (|log_2_ fold change| ≥ 0.58, adjusted *P* < 0.05). The optimal trajectory group showed a broader transcriptional response than the suboptimal trajectory group, with 470 significantly downregulated genes compared with 18 in the suboptimal group.

**Figure 4:**
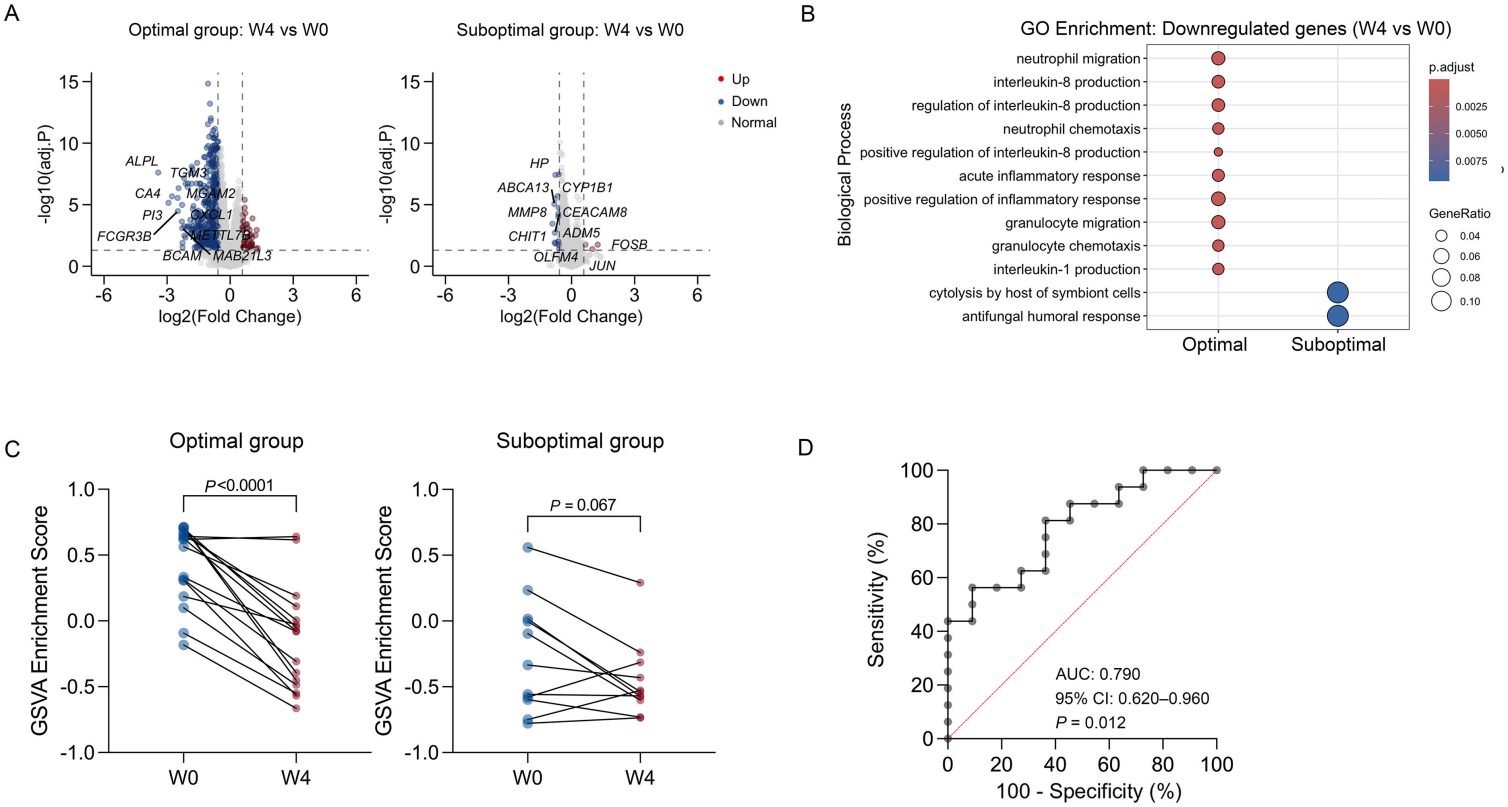
Early transcriptomic changes from baseline to week 4 according to PASI trajectory. **(A)** Volcano plots showing differential gene expression between week 4 (W4) and baseline (W0) PBMC samples within the optimal and suboptimal PASI trajectory groups. Genes with an |log_2_ fold change| ≥ 0.58 and an adjusted *P* < 0.05 were considered significant. Selected representative genes are labeled. **(B)** Gene Ontology enrichment analysis of downregulated genes identified in the optimal and suboptimal trajectory groups in the W4 versus W0 comparison. Dot size represents gene ratio, and color indicates adjusted *P* value. **(C)** GSVA enrichment scores of the neutrophil signature at baseline and week 4 in the optimal and suboptimal trajectory groups. Paired samples from the same patient are connected by lines. *P* values were calculated using paired comparisons within each group. **(D)** Receiver operating characteristic curve showing the performance of the change in neutrophil signature enrichment score from baseline to week 4 for discriminating the suboptimal trajectory. The area under the curve (AUC), 95% confidence interval, and *P* value are shown.

Among the top downregulated genes in the optimal trajectory group were *ALPL*, *CA4*, *PI3*, *CXCL1*, and *FCGR3B*, several of which are linked to neutrophil- and myeloid-associated inflammatory programs (**Figure 4A**). Gene Ontology analysis showed enrichment of innate myeloid and inflammatory processes, including neutrophil migration, IL-8 production, and acute inflammatory response, among genes downregulated in the optimal trajectory group. These changes were less evident in the suboptimal trajectory group (**Figure 4B**).

Using a mature low-density neutrophil signature, GSVA showed a larger reduction in neutrophil-related enrichment from baseline to week 4 in the optimal trajectory group, whereas the suboptimal trajectory group showed a smaller, non-significant change (**Figure 4C**). Consistent with this pattern, exploratory ROC analysis of the change in mature low-density neutrophil signature enrichment score showed moderate discrimination between the two trajectory groups (AUC 0.79, 95% CI 0.62–0.96; *P* = 0.012) (**Figure 4D**).

## Discussion

In this real-world cohort of secukinumab-treated patients with plaque psoriasis, we identified two distinct 52-week PASI response trajectories. Most patients followed an optimal trajectory with rapid and sustained improvement, while a smaller subgroup showed persistently higher disease activity. Higher baseline PASI, BMI, prior biologic exposure, multi-seasonal flare pattern, and infection-triggered flare history were associated with the suboptimal trajectory. In an exploratory PBMC transcriptomic subset, patients in the optimal trajectory showed a stronger baseline myeloid-inflammatory profile, particularly involving mature low-density neutrophil signatures, and a greater reduction in this signal by week 4.

The use of trajectory-based modeling is a key feature of this study. Psoriasis treatment response is commonly assessed using fixed timepoint endpoints, such as PASI 75, PASI 90, PASI 100, or PGA 0/1 at Week 12 and Week 52. These measures do not fully describe the pattern of response over time. By applying LCMMs, we captured longitudinal treatment heterogeneity and identified two clinically interpretable response patterns over 52 weeks. This approach may better reflect real-world treatment courses than single-visit endpoints.

Higher BMI, prior biologic exposure, and greater baseline disease severity have all been linked to less favorable treatment outcomes (23–25). Our findings extend these observations to a trajectory-based outcome. In particular, obesity may continue to influence the durability of response even during IL-17A blockade, suggesting that long-term disease control may require attention not only to biologic therapy but also to metabolic status. Prior biologic exposure may similarly identify patients with a more refractory disease course. Patients with higher baseline PASI may also require closer monitoring, stable maintenance therapy, and caution with tapering or discontinuation.

Both infection-triggered flare history and multi-seasonal flare pattern were associated with the suboptimal trajectory (26, 27). These findings suggest that previous disease instability may carry forward into the treatment course. Even under IL-17A blockade, patients with recurrent infection-related flares or multi-seasonal activity may remain prone to less stable long-term control. These variables are easy to collect in routine practice and may help identify patients who need closer follow-up.

Week 4 PASI improvement also differed between the two trajectory groups. This finding is clinically relevant because it suggests that divergence between long-term response patterns is already apparent within the first month of treatment. However, this analysis should not be interpreted as an independent prediction model, because Week 4 PASI contributed to the trajectory classification itself. Rather, it shows that early clinical response is aligned with the subsequent longitudinal course. Although Week 4 provided an early exploratory landmark for distinguishing long-term trajectories, the present study was not designed to compare the discriminative performance of multiple early follow-up visits. Future studies should assess whether later early-treatment timepoints provide improved clinical discrimination.

The transcriptomic findings provide a possible biological context for the clinical heterogeneity. At baseline, patients who later followed the optimal trajectory showed higher expression of genes related to myeloid inflammation and neutrophil-mediated immunity. They also showed stronger enrichment of mature low-density neutrophil signatures. One possible interpretation is that these patients had a more IL-23/Th17-skewed systemic inflammatory state, with greater downstream neutrophil mobilization and activation (28, 29). Such inflammation may be more sensitive to IL-17A blockade, resulting in a more rapid and sustained clinical response. Although several immune cell-associated signatures differed at baseline, the mature low-density neutrophil signature was the most consistent signal across both baseline and early-treatment analyses in this exploratory dataset.

The paired week 4 transcriptomic analysis supported this interpretation. Patients in the optimal trajectory group showed broader transcriptional downregulation and a greater decrease in neutrophil-related enrichment after secukinumab treatment. This pattern is consistent with previous reports implicating low-density neutrophils in psoriasis (30, 31). Nevertheless, these transcriptomic results should be viewed as hypothesis-generating. The sample size was small, and PBMC profiles may not fully reflect immune events in lesional skin.

This study has several limitations. The transcriptomic analysis included only 27 patients and therefore requires validation in larger independent cohorts. As an observational study, residual confounding cannot be excluded. In addition, trajectory classification and week 4 discrimination were derived from the same dataset, so the early response findings also require external validation. The transcriptomic analysis was limited to paired baseline and Week 4 PBMC samples, and longer-term serial blood sampling was not included; such data will be important for clarifying the temporal dynamics of these molecular changes. The PBMC transcriptomic results should be interpreted as exploratory and hypothesis-generating. Independent validation in external cohorts will be necessary to confirm the robustness and generalizability of the identified neutrophil-related and myeloid-inflammatory signals. We also did not have standardized baseline data on nail involvement or site-specific disease distribution across the full cohort and therefore could not evaluate these features as trajectory predictors. Finally, flare history was based on patient report and may have been affected by recall bias.

Higher baseline PASI was associated with the suboptimal trajectory, whereas stronger inflammatory and low-density neutrophil signatures were observed in the optimal trajectory group. This apparent discrepancy may imply that PBMC transcriptomes provide only an indirect view of immune activity in psoriatic skin. PASI captures skin disease burden, while PBMC profiles reflect circulating immune activity. The peripheral neutrophil-related signal may indicate an IL-17A-sensitive immune state rather than greater skin severity. Paired skin and blood transcriptomic studies are needed to clarify this relationship.

In conclusion, secukinumab-treated patients with plaque psoriasis followed two distinct 52-week PASI response trajectories. Baseline clinical features and exploratory mature low-density neutrophil signatures were associated with long-term response patterns.

## Data Availability

The datasets presented in this study can be found in online repositories. The names of the repository/repositories and accession number(s) can be found in the NCBI, accession number: GSE328820.
