# Supplementary material for: Trajectory-based profiling of 52-week secukinumab response reveals baseline clinical and transcriptomic differences in plaque psoriasis
Source: Front Immunol. 2026 Jul 31;17:1902716. doi: 10.3389/fimmu.2026.1902716 (PMC13472955; doi:10.3389/fimmu.2026.1902716)
Supplement: Supplementary file 1 [file Table1.docx]

**Supplementary Table 1** Baseline characteristics of the 27 patients included in the PBMC RNA-seq analysis, stratified by subsequent PASI response trajectory

| **Characteristics** | **Entire cohort** | **Optimal response trajectory** | **Suboptimal response trajectory** |
| --- | --- | --- | --- |
| Patients (n) | 27 | 16 | 11 |
| Age, years | 49 (38–56) | 40 (35–49) | 54 (46–59) |
| Disease duration, years | 13 (8–20) | 13 (8–20) | 13 (8–22) |
| Male sex, n (%) | 17 (63.0) | 9 (56.2) | 8 (72.7) |
| BMI (kg/m^2^) | 24.9 (23.9–26.8) | 24.4 (23.4–25.3) | 25.6 (24.5–31.4) |
| Ever smoked, n (%) | 9 (33.3) | 5 (31.2) | 4 (36.4) |
| Family history of psoriasis, n (%) | 7 (25.9) | 4 (25.0) | 3 (27.3) |
| Prior biologic exposure, n (%) | 5 (18.5) | 1 (6.2) | 4 (36.4) |
| Prior phototherapy, n (%) | 13 (48.1) | 5 (31.2) | 8 (72.7) |
| Psoriatic arthritis, n (%) | 3 (11.1) | 3 (18.8) | 0 (0) |
| Number of joint symptoms^a^, n (%) |  |  |  |
| 0 | 19 (70.4) | 10 (62.5) | 9 (81.8) |
| 1 | 6 (22.2) | 4 (25.0) | 2 (18.2) |
| ≥ 2 | 2 (7.4) | 2 (12.5) | 0 (0) |
| Number of comorbidities^b^, n (%) |  |  |  |
| 0 | 17 (63.0) | 13 (81.2) | 4 (36.4) |
| 1 | 5 (18.5) | 3 (18.8) | 2 (18.2) |
| ≥ 2 | 5 (18.5) | 0 (0.0) | 5 (45.5) |
| Flare seasonality pattern^c^, n (%) |  |  |  |
| Aseasonal | 8 (29.6) | 6 (37.5) | 2 (18.2) |
| Multi-seasonal flare | 4 (14.8) | 2 (12.5) | 2 (18.2) |
| Winter flare | 15 (55.6) | 8 (50.0) | 7 (63.6) |
| Summer flare | 0 (0) | 0 (0) | 0 (0) |
| Trigger history^d^, n (%) |  |  |  |
| Infection-triggered flare history | 7 (25.9) | 1 (6.2) | 6 (54.5) |
| Stress-triggered flare history | 1 (3.7) | 1 (6.2) | 0 (0) |
| Baseline PASI | 5.2 (4.8–9.0) | 5.0 (4.4–5.2) | 10.0 (8.5–10.6) |
| Baseline DLQI | 12 (9–16) | 11 (9–14) | 13 (7–17) |

PBMC, peripheral blood mononuclear cell; PASI, Psoriasis Area and Severity Index; BMI, body mass index; DLQI, Dermatology Life Quality Index.

Continuous variables are presented as median (interquartile range) and were compared using the Wilcoxon rank-sum test. Categorical variables are presented as number (percentage) and were compared using Fisher’s exact test.

^a^Joint symptoms included joint pain, digit pain, heel pain, back pain and stiffness.

^b^Comorbidities included cardiovascular disease, diabetes, hypertension, non-alcoholic fatty liver disease (NAFLD), hyperlipidemia, and hyperuricemia.

^c^Mutually exclusive.

^d^Multiple responses allowed.
